# Pectin supplementation accelerates post-antibiotic gut microbiome reconstitution orchestrated with reduced gut redox potential

**DOI:** 10.1093/ismejo/wrae101

**Published:** 2024-06-10

**Authors:** Rongying Xu, Ni Feng, Qiuke Li, Hongyu Wang, Lian Li, Xiaobo Feng, Yong Su, Weiyun Zhu

**Affiliations:** Laboratory of Gastrointestinal Microbiology, Jiangsu Key Laboratory of Gastrointestinal Nutrition and Animal Health, College of Animal Science and Technology, Nanjing Agricultural University, Nanjing 210095, China; National Center for International Research on Animal Gut Nutrition, Nanjing Agricultural University, Nanjing 210095, China; Laboratory of Gastrointestinal Microbiology, Jiangsu Key Laboratory of Gastrointestinal Nutrition and Animal Health, College of Animal Science and Technology, Nanjing Agricultural University, Nanjing 210095, China; National Center for International Research on Animal Gut Nutrition, Nanjing Agricultural University, Nanjing 210095, China; Laboratory of Gastrointestinal Microbiology, Jiangsu Key Laboratory of Gastrointestinal Nutrition and Animal Health, College of Animal Science and Technology, Nanjing Agricultural University, Nanjing 210095, China; National Center for International Research on Animal Gut Nutrition, Nanjing Agricultural University, Nanjing 210095, China; Laboratory of Gastrointestinal Microbiology, Jiangsu Key Laboratory of Gastrointestinal Nutrition and Animal Health, College of Animal Science and Technology, Nanjing Agricultural University, Nanjing 210095, China; National Center for International Research on Animal Gut Nutrition, Nanjing Agricultural University, Nanjing 210095, China; Laboratory of Gastrointestinal Microbiology, Jiangsu Key Laboratory of Gastrointestinal Nutrition and Animal Health, College of Animal Science and Technology, Nanjing Agricultural University, Nanjing 210095, China; National Center for International Research on Animal Gut Nutrition, Nanjing Agricultural University, Nanjing 210095, China; Research Institute of General Surgery, Jinling Hospital, Nanjing University School of Medicine, Nanjing 210095, China; Laboratory of Gastrointestinal Microbiology, Jiangsu Key Laboratory of Gastrointestinal Nutrition and Animal Health, College of Animal Science and Technology, Nanjing Agricultural University, Nanjing 210095, China; National Center for International Research on Animal Gut Nutrition, Nanjing Agricultural University, Nanjing 210095, China; Laboratory of Gastrointestinal Microbiology, Jiangsu Key Laboratory of Gastrointestinal Nutrition and Animal Health, College of Animal Science and Technology, Nanjing Agricultural University, Nanjing 210095, China; National Center for International Research on Animal Gut Nutrition, Nanjing Agricultural University, Nanjing 210095, China

**Keywords:** antibiotics, gut redox potential, microbiome reconstitution, pectin

## Abstract

Antibiotic-induced gut dysbiosis (AID) presents a big challenge to host health, and the recovery from this dysbiosis is often slow and incomplete. AID is typically characterized by elevation in redox potential, *Enterobacteriaceae* load, and aerobic metabolism. In our previous study, a pectin-enriched diet was demonstrated to decrease fecal redox potential and modulate the gut microbiome. Therefore, we propose that pectin supplementation may modulate gut redox potential and favor post-antibiotic gut microbiome reconstitution from dysbiosis. In the present study, rats with AIDwere used to investigate the effects of pectin supplementation on post-antibiotic gut microbiome reconstitution from dysbiosis. The results showed that pectin supplementation accelerated post-antibiotic reconstitution of gut microbiome composition and function and led to enhancement of anabolic reductive metabolism and weakening of catabolic oxidative pathways. These results were corroborated by the measurement of redox potential, findings suggesting that pectin favors post-antibiotic recovery from dysbiosis. Pectin-modulated fecal microbiota transplantation accelerated the decrease in antibiotics-elevated redox potential and *Enterobacteriaceae* load similarly to pectin supplementation. Moreover, both pectin supplementation and Pectin-modulated fecal microbiota transplantation enriched anaerobic members, primarily from *Lachnospiraceae* orchestration with enhancement of microbial reductive metabolism in post-antibiotic rats. These findings suggested that pectin supplementation accelerated post-antibiotic gut microbiome reconstitution orchestrated with reduced gut redox potential and that the effect of pectin on redox potential was mediated by remodeling of the intestinal microbiota.

## Introduction

As a crucial part of modern medicine, antibiotics are powerful tools in fighting against bacterial infections, but antibiotic use often leads to remarkable perturbation of the gut microbiome [1]. Unfortunately, this disturbance is also often associated with health complications, for example, prolonged pathogen susceptibility [2, 3], inflammatory disease, diarrhea [4], and other metabolic disorders [5–7]. This antibiotic-induced gut dysbiosis (AID) is typically characterized by increases in aerobic bacterial metabolism and redox potential and abundance of *Enterobacteriaceae* [8–11]. Post-antibiotic microbiome reconstitution is often slow and variable [12]. Dietary fibers of different types can modulate the gut chemical environment and microbial community through fermentation, which is favored in an anaerobic gut environment, suggesting the potential of dietary fibers to thermodynamically alleviate gut dysbiosis [13, 14]. A recent study has demonstrated that compared with a glucose diet, a fiber cocktail may protect against antibiotic-induced gut microbiome dysbiosis by modulating gut redox potential [8], the importance of which in defense against pathogens was recognized as early as the 1960s [15]. To our knowledge, however, at the time of this report this efficacy had not yet been assessed at the scale of a single type of fiber.

Pectin is a common complex polysaccharide found in plant cell walls that consists mainly of α-1,4–linked galacturonic acid residues and has been recognized as an attractive prebiotic with beneficial effects on host health. Pectin serves as a fermentable nutrient for the complex microbial community in the hindgut and in parallel drives microbial fermentative metabolism of carbohydrate substrates. Microbial fermentation drives oxygen consuming reactions in the gut [16], which are vital for maintaining an anaerobic gut environment with a low redox potential. Our previous research with pigs revealed that pectin supplementation decreased fecal redox potential and through colon metatranscriptome analysis unveiled that pectin supplementation may decrease the relative abundances of genes involved in microbial electron transport [17]. High levels of electron transport chain activity are often associated with active aerobic metabolism [8]. Thus, we proposed that pectin may help to decrease antibiotic-elevated gut redox potential and improve post-antibiotic reconstitution of the gut microbiome. Based on this proposal, we sought to elucidate the underlying mechanism for the modulation of gut redox potential by pectin.

Diet modulates the gut chemical environment, a process involving various aspects, such as chemical characteristics, host response, and microbial activities. Antioxidant properties of dietary constituents, like phenolic compounds, vitamins, and dietary fibers, have been reported extensively to defend against oxidative stress and improve gut redox status [18–21]. Host redox status may affect gut redox signals [22]. In addition, colonized microbiota are also important modulators for redox dynamics in the gut lumen [9]. Therefore, when exploring how pectin influences the redox state of the gut lumen, these factors should be taken into consideration.

In this study, we investigated the impact of pectin supplementation on post-antibiotic recovery of the gut chemical environment and its microbiome composition and function in rats. Pectin accelerated reduction of antibiotic-elevated redox potential and post-antibiotic reconstitution of gut microbiome composition and function. Further, we explored how pectin modulated gut redox potential after antibiotic treatment. To this aim, we assessed changes in the host phenotype and examine the impacts of pectins with different antioxidant ability and pectin-modulated gut microbiota on post-antibiotic recovery, respectively. The findings of the current study provide new insights for developing therapeutic strategies to relieve AID and identify potential targets for improvement of the gut microbiota.

## Materials and methods

### Rats

Four-week old male Sprague–Dawley rats (average initial weight 120 g) were purchased from Cavens Biogle (Suzhou, China) and allowed to acclimatize to the animal facility environment for 1 week prior to the experiments. All rats were singly housed and had ad libitum access to food and water. Rats were fed with a standard chow diet (Table S1) (http://www.jsxtsw.com/320/, Xietong Shengwu Co., Nanjing, China). The dietary fiber was mainly derived from corn, wheat, and soybean meal.

### Antibiotic-induced gut dysbiosis rat models

For antibiotic treatment, rats were orally gavaged with either 2 ml of autoclaved deionized water or 2 ml of an antibiotic cocktail daily for 5 days as previously described [9]. The antibiotic cocktail consisted of 1 mg/ml ampicillin (Sangon Biotech, Shanghai), 5 mg/ml vancomycin (Sangon Biotech, Shanghai), 10 mg/ml neomycin (Aladdin, Shanghai), and 10 mg/ml metronidazole (Sangon Biotech, Shanghai). The immediate impact of antibiotic treatment on the colon microbiome was assessed in 1 group of rats euthanized after 5 days of antibiotic exposure (ABX group; *n* = 6) compared with the untreated controls(CON group; *n* = 6).

### Animal experiments

**Figure 1:**
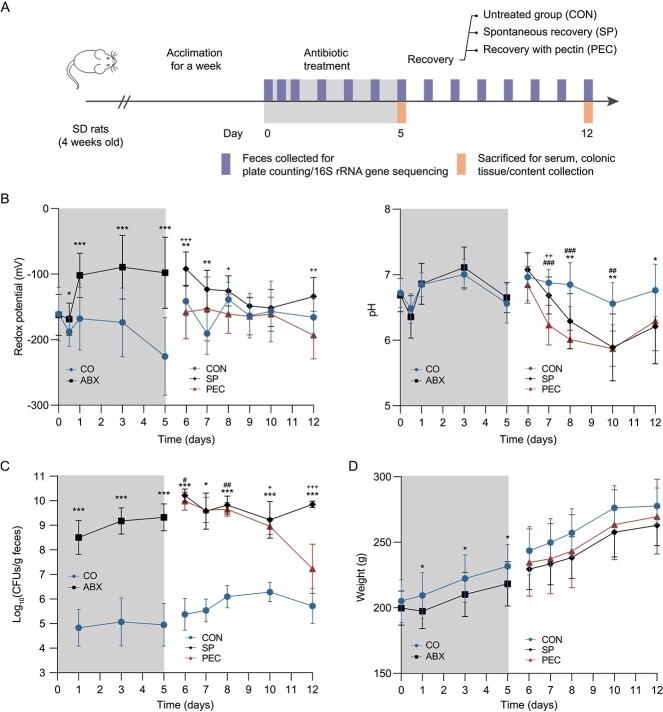
**Pectin supplementation enhances the post-antibiotic recovery from antibiotic-induced gut dysbiosis (AID). A** Experimental design (*n* = 6). Dynamic changes in **B** redox potential and pH values, **C**  *Enterobacteriaceae* load and **D** body weight in rat during and after antibiotic treatment. The stage of antibiotic treatment was noted with a grey background, and the significant difference between the control (CON) and the antibiotic-treated (ABX) groups was tested with the Student *t* test. ^*^P < .05, ^*^^*^P < .01, ^*^^*^^*^P < .001. In recovery stage, statistical significance among the untreated (CON), spontaneous recovery (SP) and pectin supplemented (PEC) groups was determined using one-way ANOVA. Three symbols (^*^, #, and +) indicate the significance of SP vs CON, PEC vs CON and PEC vs SP, respectively. Day 6 is 1 day (24 hr) since the timing of initiation of pectin in the treatment group.

One day after the final gavage of antibiotic cocktail, the untreated rats were assigned to an untreated CON group (*n* = 6), and the remaining antibiotic-treated rats (*n* = 12) were randomly subdivided into 2 post-antibiotic intervention groups, the spontaneous recovery (SP; *n* = 6) and pectin supplementation (PEC; *n* = 6) groups (Fig. 1A). For pectin treatment, rats were given apple pectin (galacturonic acid content + 65.0%; Yuanye, Shanghai, China) in their drinking water at a concentration of 15 mg/ml. The CON group rats did not receive antibiotics or any other treatment throughout the experiment. Fresh fecal samples were collected over multiple time points: before antibiotic treatment (day 0), midpoint of antibiotic treatment (day 3), endpoint of antibiotic treatment (day 5), 1 day post-gavage (day 6), 3 days post-gavage (day 8), 5 days post-gavage (day 10), and 7 days post-gavage (day 12). Finally, all of the rats were euthanized by CO_2_ asphyxiation, and blood samples, colonic tissues, and digesta were collected. Blood glucose levels were determined with a blood glucose meter (Sinocare, Changsha, China). Colon samples were immediately snap-frozen and transferred for storage at −80°C until further processing.

All animal experiments of this study were strictly performed with protocols approved by the Ethical Committee of Nanjing Agricultural University in compliance with the Regulations for the Administration of Affairs Concerning Experimental Animals of China.

### Intervention experiments of pectins with different antioxidant capacities

Six commercially available pectins were purchased (Table S2). Antioxidant capacity measurement methods such as 2,2-diphenyl-1-picrylhydrazyl (DPPH) radical scavenging activity, 2,2'-azinobis (3-ethylbenzothiazoline-6-sulfonic acid) (ABTS) radical scavenging activity, and ferric reducing power were applied to evaluate the antioxidant performance of pectin. In consideration of their antioxidant performances collectively, we screened out 3 pectins with high-, middle-, and low-antioxidant performance and marked them as as HP, MP, and LP, respectively. The pectin used in the MP group was the same as that used in the experiment described above. A pilot experiment was performed to evaluate the effects of different pectins on the gut chemical environment. Rats were randomly divided into 4 groups on the basis of pectin supplementation: the CON group (*n* = 6) and the HP, MP, and LP groups (*n* = 6 each). In detail, pectins were supplemented into the drinking water (15 mg/ml) of rats in the HP, MP, and LP groups but not in the CON group. All interventions continued for 8 days. During the experiment, fresh fecal samples were collected daily for determining redox potential and pH measurements.

Pectins with high- and low-antioxidant abilities were applied to ABX-treated rats. The manner in which the antibiotics induced gut dysbiosis in rats was consistent with the above. After 5 days of antibiotic exposure, ABX-treated rats were randomly divided into the following 3 groups: the spontaneous recovery group (SP; *n* = 7), and the HP (*n* = 7) and LP (*n* = 7) supplementation groups. The rats in the CON group (*n* = 7) did not receive antibiotics or any other treatment throughout the experiment. Fresh fecal samples were collected over the recovery stage for measuring redox potential and plate counting of *Enterobacteriaceae*. The fecal samples collected on the last day were immediately snap-frozen and transferred for storage at −80°C for further 16S rRNA gene sequencing analysis.

### Fecal microbiota transplantation

Fecal microbiota transplantation was performed according to the literature [23]. For microbiota suspension preparation, fresh fecal samples of donor rats in the CON group (*n* = 6) and the MP group (*n* = 6) were collected and pooled separately at the end of the pilot experiment on pectin intervention, followed by dilution with chilled phosphate-buffered saline solution (100 mg feces/1 ml buffer). Then the samples were mixed well and centrifuged at 800 *g* for 5 minutes. The supernatant was collected and stored in 20% sterile glycerol at −80°C until transplantation. Male Sprague–Dawley rats underwent a 7-day adaptation stage and then were gavaged with a cocktail of antibiotics for 5 consecutive days as described above. Subsequently, recipient post-antibiotic rats gavaged daily with a 2-ml fecal microbial suspension from the CON and MP rats were divided into the normal fecal microbiota transplantation (FMT) group (*n* = 3) and the pectin-modulated fecal microbiota transplantation (P-FMT) group (*n* = 3), respectively. Fresh fecal samples were collected over multiple postantibiotic time points (days 6, 8, 9, 10, and 12) for further analysis. On day 12, the rats were euthanized, and relevant samples (e.g., serum and colonic contents) were collected in line with the methods described above.

### Redox potential and pH measurements

The method of fecal redox potential measurement has been previously described in the literature [17]. Briefly, fresh fecal samples were immediately collected for redox potential measurement within 2 minutes using an ST300/B oxidation-reduction potential electrode, and all measurements were performed inside an anaerobic chamber under anaerobic conditions. For fecal pH measurements, fresh fecal samples were collected and pH was determined using a hand-held pH meter (Hanna, Italy).

### Plate counting

Serial dilution of 0.1 mL of a homogenized fecal sample was performed with sterile water and 1:10 increments, and a 0.1-mL diluted sample was inoculated onto a sterile MacConkey agar plate and then incubated for 24 hours at 37°C to culture the *Enterobacteriaceae*. The colony-forming units on each MacConkey agar plate were counted.

### DNA isolation, 16S rRNA gene sequencing, and processing

Microbial DNA was isolated from frozen fecal samples using the E.Z.N.A. Stool DNA Kit (Omega Bio-tek, Norcross, GA, United States) according to the manufacturer’s protocols. We performed 16S rRNA gene amplicon sequencing using the custom barcoded primers 341F 5′-CCTACGGGNGGCWGCAG-3′ and 806R 5′-GGACTACHVGGGTATCTAAT-3′ targeting the V3–V4 region of the gene. Sequencing was conducted on a MiSeq platform (Illumina, San Diego, CA, United States) with paired-end 250-bp reads. The sequences were clustered into operational taxonomic units (OTUs) at 97% nucleotide identity using Usearch (version 10) [24]. Alpha diversity indexes (richness and Shannon index) were calculated using the R (v4.0.2) software packages vegan (v2.5–6) [25] and picante (v1.8.2). Principal coordinates analysis (PCoA) of OTUs was performed based on the Bray-Curtis metric with the R (v4.0.2) software package vegan (v2.5–6) [25] and visualized with the R package ggplot2 (v3.3.6) to show dynamic shifts of microbial communities over the whole experiment. Based on the Kyoto Encyclopedia of Genes and Genomes (KEGG) database (v90.0) [26], PICRUSt [27] was applied to predict the functional profiling of microbial communities. After differential analysis was conducted between the groups with the Wilcoxon rank-sum test in R software (v4.2.1), the genus or pathway in treated groups that was significantly different from the CON group in relative abundance was identified as the “unrestored” genus or pathway. The accumulative relative abundances of unrestored genera/pathways in treated groups were summarized within each timepoint during post-antibiotic recovery. The CON groups for SP and PEC at D8 and D10 were the CON group at D6 and D12, respectively. The heatmap was plotted by use of https://www.bioinformatics.com.cn (last accessed on 10 May 2023), an online platform for data analysis and visualization.

### Metagenome sequencing

Microbial DNA extraction and metagenomic shotgun sequencing were performed according to our previous study [17]. Briefly, DNA samples of rat colon microbiota were sequenced in the HiSeq X instrument (Illumina, San Diego, CA). The raw data underwent quality trimming using Trimmomatic (v0.36) [28] and BWA (v0.7.17) [29] to remove adaptors, low-quality reads, and host–genome contamination. Then, clean reads were assembled using MEGAHIT (v1.1.2) [30] with a minimum-contig-length 500 parameter. The open reading frames of assembled contigs were predicted using Prodigal (v2.6.3) [31] and aggregated into a nonredundant microbial gene set after clustering by CD-HIT (v4.6.7) [32].

### Taxonomy and function analysis

DIAMOND (v0.9.22), with the standard protein Basic Local Alignment Search Tool (BLASTP) was applied to perform a taxonomic assessment of the colon microbiota based on the NCBI nonredundant database (https://www.ncbi.nlm.nih.gov/refseq/about/nonredundantproteins/). The nonredundant gene set was mapped to the KEGG (v90.0) [26] database using KofamScan (v1.2.0) with the HMMSEARCH (v3.3) package [33]. The abundances of microbial taxa, KEGG orthology (KO), and pathways were normalized to transcripts per million. Alpha diversity indexes (richness and Shannon index) were calculated using mothur (v1.44.2) [34]. The PCA analysis was performed with the package vegan (v2.5–6) [25] and visualized with the package ggplot2 (v3.3.6) from R (v4.0.2) software. The heatmap was plotted by using https://www.bioinformatics.com.cn (last accessed on 20 May 2023).

### Histological evaluation

Collected colon tissues were fixed in 4% paraformaldehyde and embedded in paraffin. Paraffin-embedded tissues were sectioned (4 μm thickness) and subjected to hematoxylin and eosin staining. Then, histological images were obtained using a light microscope (Axio Scope.A1, Carl Zeiss, Oberbochen, Germany) under identical conditions and at the same magnification. Histological scores were evaluated as previously described [35], and the criteria are described in Table S3.

### Nitrate measurement

The nitrate levels of rat feces from post-antibiotic groups were measured using rat nitrate enzyme-linked immunosorbent assay kits following the manufacturer’s instructions (Qiyuan, Shanghai, China). The intensity was detected at a wavelength of 450 nm using a microplate reader (Multiskan GO; Thermo Scientific, MA, United States).

### Serum reactive oxygen species determination

Blood samples were centrifuged at 900 g at 4°C for 10 minutes to obtain serum for the determination of reactive oxygen species (ROS). Serum ROS levels were measured by an enzyme-linked immunosorbent assay kit (Shanghai Yiyan Biotechnology Co. Ltd, Shanghai, China) according to the manufacturers’ protocols. The intensity was detected at a wavelength of 450 nm using a microplate reader (Spectramax M2; Molecular Devices, Sunnyvale, CA, United States).

### Antioxidant assays

The ABTS•^+^–scavenging activity of 6 commercially available pectins was evaluated with the ABTS scavenging assay test kit (Solarbio, Beijing, China) according to the manufacturer’s protocols [36]. Briefly, 190 μl ABTS•^+^ working solution was added to 10 μl of sample and incubated at room temperature for 6 minutes in the dark. The absorbance was measured at 405 nm. The ABTS radical scavenging rate percentage was calculated according to the following formula: [A_s0_ − (A_s1_ − A_s2_)]/A_s0_ × 100, where A_s0_ is the absorbance of the CON group (dH_2_O instead of sample solution), A_s1_ is the mixed solution absorbance of the sample and ABTS•^+^, and A_s2_ is the absorbance of the sample solution alone.

The DPPH•-scavenging activity of pectins was determined with the DPPH scavenging assay test kit (Solarbio, Beijing, China). Briefly, 190 μl DPPH working solution was added to 10 μl of sample and incubated at room temperature for 30 minutes in the dark. The absorbance was measured at 515 nm. The DPPH radical scavenging rate was calculated according to the following formula rate (%) = [A_s0_ − (A_s1_ − A_s2_)]/A_s0_ × 100, where A_s0_ is absorbance of the CON group (dH_2_O instead of sample solution); A_s1_ is the mixed solution absorbance of the sample and the DPPH solution; and A_s2_ is the absorbance of the sample solution alone.

Ferric reducing activity of pectins was evaluated by using the FRAP assay kit (Yuanye, Shanghai, China) according to the manual instructions. Briefly, 264 μl FRAP working solution was added to 30 μl of sample and incubated at 37°C for 30 minutes. The absorbance was measured at 593 nm. The results were expressed as Fe^2+^ quivalents.

### Statistical analysis

The GraphPad Prism (version 9) and R (v4.2.1) software were used. The significant differences between the treated and CON groups were tested by one-way ANOVA with SPSS (version 21.0, IBM, Armonk, NY, United States) followed by LSD’s multiple comparisons test. The differences in relative abundance between groups were tested by Wilcoxon rank-sum test by R (v4.2.1) software. The data were presented as the means ± SD in each group. The differences were considered to be significant at *P* < .05. Correlations between datasets were calculated using Spearman’s rank correlation by the package “psych (v 2.2.9)” of R (v 4.2.1) software. Besides, specific details of the statistical analyses for all experiments were displayed in the figure legends.

## Results

### Pectin supplementation accelerates post-antibiotic recovery from AID

The redox potential (Eh) and pH in freshly fecal pellet of rats were important environment factors and were measured daily in parallel during the experiment. Rats that underwent antibiotic treatment had significantly increased fecal Eh levels compared with the CON rats (Fig. 1B, *P* < .001, *t*-test). Compared to spontaneous post-antibiotic recovery, pectin supplementation induced a faster recovery in rat fecal redox potential and even achieved a significant lower level in the end of the administration, but no significant differences in pH values of rats were observed between the 2 groups. The striking increase of *Enterobacteriaceae* was observed after antibiotics treatment (Fig. 1C), indicating the evident disruption of the fecal microbial community structure. In D12, *Enterobacteriaceae* load of spontaneous recovery (SP) group was significantly higher than that of untreated (CON) (*P* < .001, *t*-test) and pectin supplementation (PEC) groups (*P* < .001, *t*-test), whereas no significant difference was noted between the PEC and CON groups. During antibiotics treatment, the mean body weight of rats upon antibiotics exposure (ABX) were significantly lower than that of the control (*P* < .05, *t*-test). But no significant differences in weight of rats were observed among the CON, SP, and PEC groups during recovery (Fig. 1D). The effects of post-antibiotic recovery treatments on average daily feed intake and blood glucose level were assessed (Fig. S1A-B). There were no significant differences in average daily feed intake and glucose of rats among the CON, SP, and PEC groups.

### Dynamics of post-antibiotic microbiome reconstitution in rat feces of recovery groups

**Figure 2:**
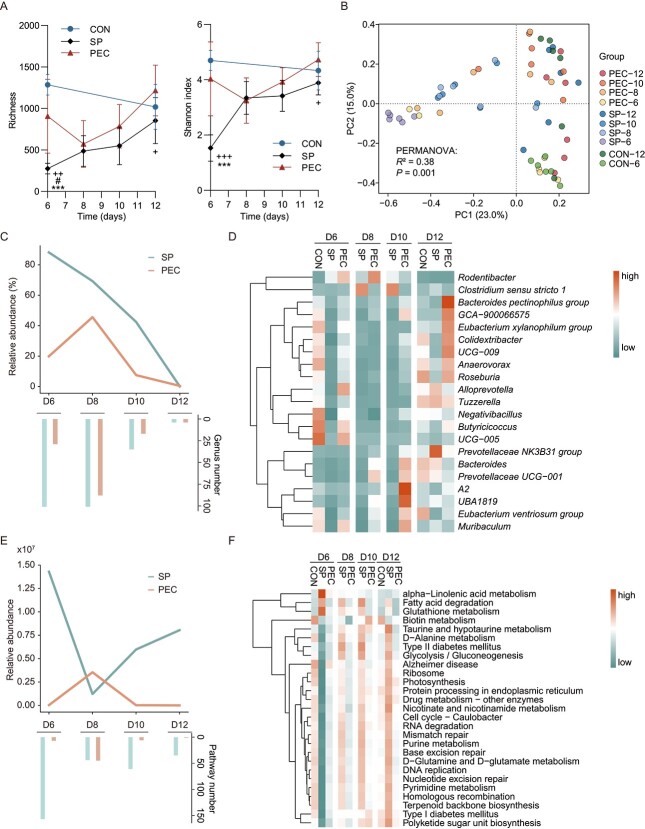
**Dynamic effects of pectin supplementation on the post-antibiotic reconstitution of fecal microbiome in rats. A** Dynamic changes in α-diversity of the fecal microbiota in recovery stage. Statistical significance was determined using one-way ANOVA. Three symbols (^*^, #, and +) indicate the significance of SP vs CON, PEC vs CON and PEC vs SP, respectively. **B** Principal-coordinates analysis (PCoA) of fecal microbiome among CON, SP and PEC rats along with time after antibiotics treatment. PCoA was performed based on the Bray–Curtis metric. **C**, **E** The abundance and number of unrestored genera/pathways. Genus/pathway in treated groups that significantly different from the CON was identified as “unrestored”. Wilcoxon rank-sum test, *P* < .05. **D**, **F** Genera/pathways significantly altered by antibiotics in feces, which became indistinguishable to controls earlier in PEC than in SP. In the heatmap, relative abundances of genera/pathways were normalized by a *z*-score approach. Wilcoxon rank-sum test, *P* < .05. The functional prediction in **E** and **F** was performed by PICRUSt.

The impact of the pectin supplementation on reconstitution of the indigenous rat fecal microbiome community following antibiotic treatment was determined by 16S rRNA gene sequencing. Of the post-antibiotic interventions, PEC was more efficient than SP in restoring fecal bacterial richness and Shannon index to that observed in the CON, with alpha diversity becoming distinguishable to CON within 7 days following PEC (Fig. 2A, *P* < .05, *t*-test). The principal coordinates analysis (PCoA) plot revealed that PEC-induced microbiome reconstitution was faster than that in SP to approach to fecal microbiota composition in the CON group (Fig. 2B).

To get insights into the underlying mechanisms of PEC on improving post-antibiotic microbiome reconstitution, microbiota profiling was performed to compare the composition of fecal microbiota in different interventions during post-antibiotic recovery. The number and mean accumulative relative abundance of unrestored genera and pathways, which have significant difference in relative abundance with the CON group, from rats were calculated within each time point during post-antibiotic recovery. The results demonstrated that both number and mean accumulative relative abundance of unrestored genera and pathways in PEC rats were lower than that of SP rats over most time (Fig. 2C and E). Of the genera altered in fecal relative abundance by antibiotics, we identified 21 that became indistinguishable to control levels faster in the PEC recovery group than the SP (Fig. 2D and Table S4). As observed, the majority of PEC-elevated genera belonged to the *Lachnospiracea* family, including the [*Bacteroides*] *pectinophilus* group, GCA-900066575, and [*Eubacterium*] *xylanophilum* group. Likewise, multiple pathways were identified that were restored faster in PEC than in spontaneous recovery. In total, 27 pathways, the majority of which were related to metabolism, were restored in the PEC recovery group, but not in the spontaneous group (Fig. 2F and Table S5). The restored pathways included glutathione metabolism, nicotinate and nicotinamide metabolism, and d-glutamine and d-glutamate metabolism.

### Pectin improves the post-antibiotic reconstitution of microbiome in rat colon

The colon is the major site for pectin fermentation. Therefore, we speculated that the colon microbiome might play an important role in promoting post-antibiotic gut microbiome reconstitution. Four samples per group were randomly selected for metagenome sequencing and generated a total of 2 038 236 596 reads, with 101 911 830 ± 5 438 642 reads (mean ± SEM) per sample (Table S6). After quality control and removal of host genes, a total of 2 028 252 216 reads were retained, with 101 412 611 ± 5 420 799 per sample. After de novo assembly, 3 267 859 contigs were generated (N50 length of 17 190 ± 9483 bp), with 163 393 ± 25 788 per sample.

**Figure 3:**
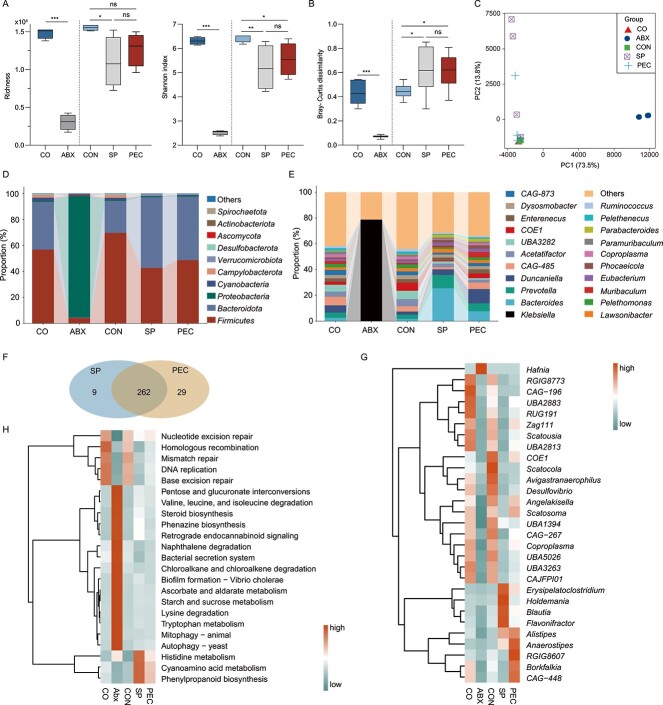
**Pectin supplementation improves the post-antibiotic reconstitution of rat colon microbiome following antibiotic treatment.**  *n* = 4 per group. **A** α-diversity and **B** β-diversity of the colon microbiota. One-way ANOVA, ^*^P < .05, ^*^^*^P < .01, ^*^^*^^*^P < .001; ns, no significant. **C** Principal-coordinates analysis (PCoA) of colon microbiome in rats using Bray–Curtis metric. Colon microbiota composition at **D** phylum and **E** genus levels. **F** Venn plot of the number of restored genera in SP and PEC groups. Genus was significantly altered by antibiotics, which became indistinguishable from controls and was identified as “restored”. **G, H** Heatmap analysis of the restored genera/pathways specific to PEC. Each column in the heat map represents one group, and each row represents one genus/pathway. The relative abundance of the genus/pathway was normalized by the *z*-score. Wilcoxon rank-sum test, *P* < .05.

After 5-day antibiotic administration, a reduction in alpha diversity (richness and Shannon index) (Fig. 3A) and a Bray–Curtis dissimilarity (Fig. 3B) were also observed in the colon metagenome. At the end of the recovery stage, the colonic microbial richness of the SP group was still significantly lower than that of the CON group, whereas the richness of the PEC group was indistinguishable from that of the CON group (*P* > .05). The PCoA plot also exhibited that PEC rats had the lowest distance compared with control rats, suggesting the similarity of colon microbiomes between PEC and CON rats (Fig. 3C). At the phylum level (Fig. 3D), antibiotic treatment resulted in the predominance of *Proteobacteria* (93.5%) in the colon metagenome, and at the end of recovery, that predominance disappeared both in the SP and PEC groups. The relative abundance of the phylum *Cyanobacteria* in PEC rats rather than SP rats became indistinguishable to CON rats (Fig. S2, *P* > .05). At the genus level (Fig. 3E), antibiotic treatment resulted in the predominance of *Klebsiella* (78.6%) in the colon metagenome, and in the end of recovery, the *Klebsiella* predominance disappeared both in the SP and PEC groups. In the CON group rat colons, the predominant genera were *UBA3282* (6.4%), *COE1* (6.3%), and *Acetatifactor* (5.7%); whereas in the SP group, the predominant genera were *Bacteroides* (25.5%), *Prevotella* (10.3%), and *Duncaniella* (4.4%); in PEC, the predominant genera were *Duncaniella* (11.1%), *Bacteroides* (7.6%), *Prevotella* (6.2%), *CAG-485* (5.0%), *Muribaculum* (4.3%), and *COE1* (3.6%).

Of the restored genera (becoming indistinguishable with the CON group), 29 and 9 genera were specific to the PEC and SP rats, respectively, suggesting the role of these specific restored genera in PEC recovery (Fig. 3F). The correlation analysis showed that most of these genera and species were significantly negatively correlated with the *Enterobacteriaceae* load (Fig. S3). The majority of the PEC-specific restored (bloomed) genera belonged to *Desulfovibrionaceae*, *Gastranaerophilaceae*, and *Lachnospiraceae* (Fig. 3G, Table S7). In KEGG pathways, 23 pathways only restored in the PEC recovery group, and mostly belonged to Metabolism (level-1 category) (Table S8). Compared with the CON group, the relative abundance of catabolic oxidative pathways such as pentose and glucuronate interconversions, starch and sucrose metabolism, and lysine degradation pathways exhibited elevation in SP, but not in PEC (Fig. 3H).

### Pectin supplementation represses oxidative metabolism and increases reductive metabolism in colon microbiome of post-antibiotic recovery rats

**Figure 4:**
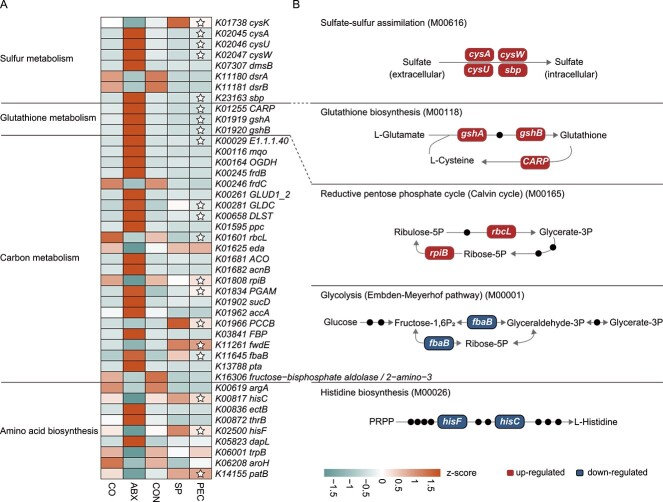
**PEC represses oxidative metabolism and increases reductive metabolism in colon microbiome of post-antibiotic recovery rats. A** Heatmap of KO genes associated with oxidative- and reductive- metabolism in colon metagenome. The grid with star indicates KO gene specifically restored in PEC. KO gene was significantly altered by antibiotics, which became indistinguishable from controls and was identified as “restored.” The relative abundance of the KO gene were normalized by the *z*-score. Wilcoxon rank-sum test, *P* > .05. **B** representative KO genes that appear in **A** are shown in pathway modules modified from KEGG pathway maps like “sulfur metabolism,” “glutathione metabolism,” “carbon metabolism,” and “biosynthesis of amino acids.” Each box in a pathway represents a KO gene.

To evaluate whether post-antibiotics microbial reconstitution orchestrated with changes in redox-associated metabolic signatures in the gut microbiome, the KO function compositions of SP and PEC rats were determined. In total, 108 KOs were observed to be restored specific to PEC (Table S9). Increased KOs involved in catabolic oxidative metabolism, including glycolysis (M00001) and histidine biosynthesis (M00026), were more associated with the SP group (Fig. 4, Table S10). In addition, PEC recovery was more associated with increased KOs assigned to anabolic reductive metabolism, such as sulfate-sulfur assimilation (M00616), glutathione biosynthesis (M00118), and reductive pentose phosphate cycle (M00165). For example, compared with the CON group, the sulfate/thiosulfate transport system ATP-binding protein (*cysA*/*EC:7.3.2.3*), permease protein (*cysW*/*cysU*), substrate-binding protein (*sbp*), glutamate cysteine ligase (*gshA*/*EC:6.3.2.2*), glutathione synthase (*gshB*/*EC:6.3.2.3*), leucyl aminopeptidase (*CARP*/*EC:3.4.11.1*), ribulose-bisphosphate carboxylase large chain (*rbcL*/*EC:4.1.1.39*), and ribose 5-phosphate isomerase B (*rpiB*/*EC:5.3.1.6*) involved in anabolic reductive metabolism were significantly lower in SP rats, whereas these KOs in PEC rats were indistinguishable from those in control rats. In contrast, fructose-bisphosphate aldolase (*fbaB*/ *EC:4.1.2.13*), 2,3-bisphosphoglycerate-dependent phosphoglycerate mutase (*PGAM*/*EC:5.4.2.11*), imidazole glycerol-phosphate synthase subunit HisF (*hisF*/*EC:4.3.2.10*), and histidinol-phosphate aminotransferase (*hisC*/*EC:2.6.1.9*) involved in catabolic oxidative metabolism were significantly higher in the colon of SP rats. These data were consistent with pectin supplementation-associated gut redox potential reduction in post-antibiotic recovery. However, no significant difference was observed in the electron transport chain (ETC) upon PEC (Fig. S4), though it has been reported that fiber could reduce usage of ETC to protect from AID [8].

### PEC accelerates reduction of post-antibiotic gut redox potential not by altering host-associated factors

PEC had no significant post-antibiotic effects on colonic morphology (Fig. S5A and S5B). Host-derived redox-active immune molecules such as ROS or nitrate are known to be associated with antibiotic-induced gut redox imbalance [9], whereas at the time of this report it was unknown whether these molecules were related to PEC-accelerated reduction of gut redox potential. In the present study, the levels of electron acceptors, including nitrate, were observed to be no different in the treated compared with the control rats (Fig. S5C). Serum ROS concentrations were measured and also revealed no significant differences between the groups (Fig. S5D). Collectively, these data provided evidence that PEC did not alter host-associated factors involved in modulating gut redox status.

### Pectins with different antioxidant abilities exert analogous effects on gut redox potential and post-antibiotic microbiome reconstitution

**Figure 5:**
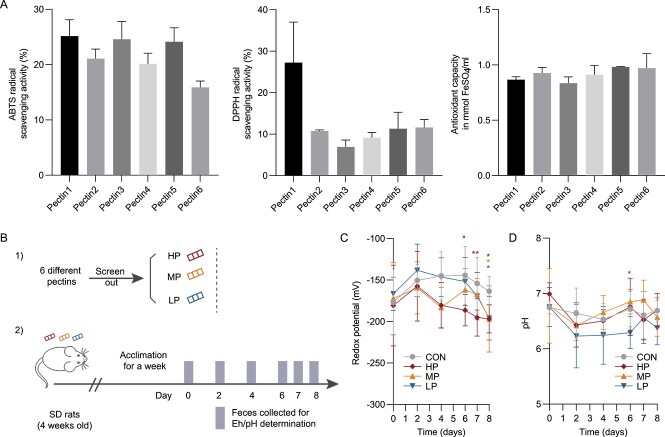
**Effects on rat fecal redox potential by pectins screened out with different antioxidative activities. A** Evaluation of antioxidant activity of pectins with indices like the ABTS, DPPH, and FRAP assay. ABTS, 2,2′-azinobis-(3-ethylbenzothiazoline-6-sulfonic acid); DPPH, 2,2-diphenyl-1-picrylhydrazyl; FRAP, ferric reducing ability. Information regarding pectins (pectin 1–6) is listed in Table S2. **B** Experimental design (*n* = 6). HP (pectin 1), MP (pectin 5), and LP (pectin 6) indicate groups treated by pectins with high, middle, and low antioxidative ability, respectively. **C** Redox potential and pH values in pectin supplemented groups throughout the rat experiment. Asterisks indicate significance in any group versus the control group. One-way ANOVA, ^*^P < .05, ^*^^*^P < .01.

Antioxidant properties of dietary contents have been reported to be associated with gut redox balance [21]. However, it is still unclear whether the antioxidant ability of pectin itself could affect gut redox potential and post-antibiotic microbiome reconstitution. Thus, we screened out three commercially available pectins with high-, middle-, and low- antioxidant abilities according to ABTS, DPPH, and FRAP assays and marked them as HP (pectin 1), MP (pectin 5), and LP (pectin 6), respectively (Fig. 5A and B). The fecal redox potential and pH in the control and MP rats were compared (Fig. 5C and D). The results demonstrated that all of these pectins could decrease the redox potential of rat feces by day 8 of the experiment, whereas no significant difference in pH was observed between the MP groups and the CON group.

**Figure 6:**
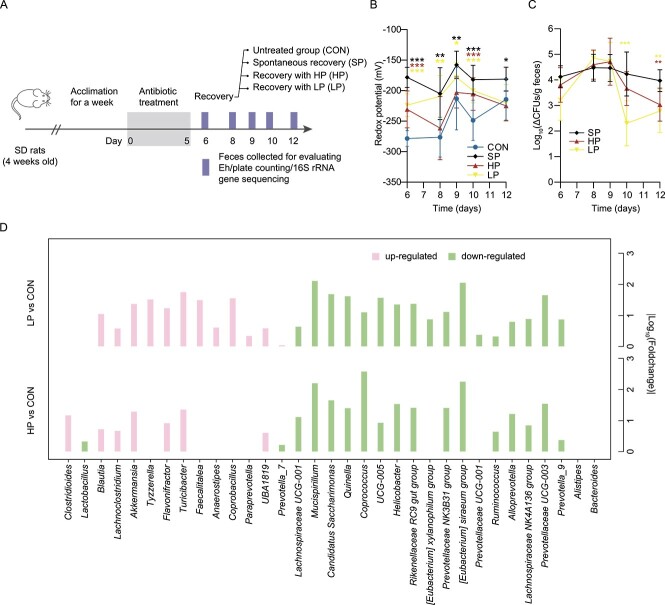
**Analogous effects were exerted on post-antibiotic recovery from AID by pectins with different antioxidative activities. A** Experimental design (*n* = 7). HP and LP indicate pectins with high and low antioxidative activity, respectively. **B** Redox potential value in post-antibiotic rat feces. Asterisks indicate significance in any group versus the control group. One-way ANOVA, ^*^P < .05, ^*^^*^P < .01, ^*^^*^^*^P < .001. **C** Effects of HP and LP on reduction of *Enterobacteriaceae* load after antibiotics treatment. Δ means the colony-forming units (CFU) of treated rats minus the average of control rats. Asterisks indicate significance in the pectin-treated (MP) group-+ versus the SP group. **D** Modulation of HP and LP on rat fecal microbiota composition by day 12. Significantly differential genera in groups versus the control group were identified.

We used ABX-rats to further assess the effects of the antioxidant ability of pectin on post-antibiotic recovery (Fig. 6A). These data showed that both HP and LP significantly decreased redox potential and *Enterobacteriaceae* abundance in post-antibiotic rat feces by day 12, indicating that pectins with different antioxidant ability impacted gut redox potential similarly (Fig. 6B and C). Moreover, in post-antibiotic microbiome reconstitution, HP and LP exerted comparable influences on microbial composition in rats by day 12 based on the results of 16S rRNA gene sequencing (Fig. 6D). In total, 31 genera with significantly different relative abundances between post-antibiotic treated groups and the CON group were identified, and 21 of them were regulated in parallel in HP and LP. For example, the *Blautia*, *Akkermansia*, and *Turicibacter* genera were enriched both in the HP and LP groups.

### Pectin-modulated intestinal microbiota accelerates post-antibiotic recovery from AID

**Figure 7:**
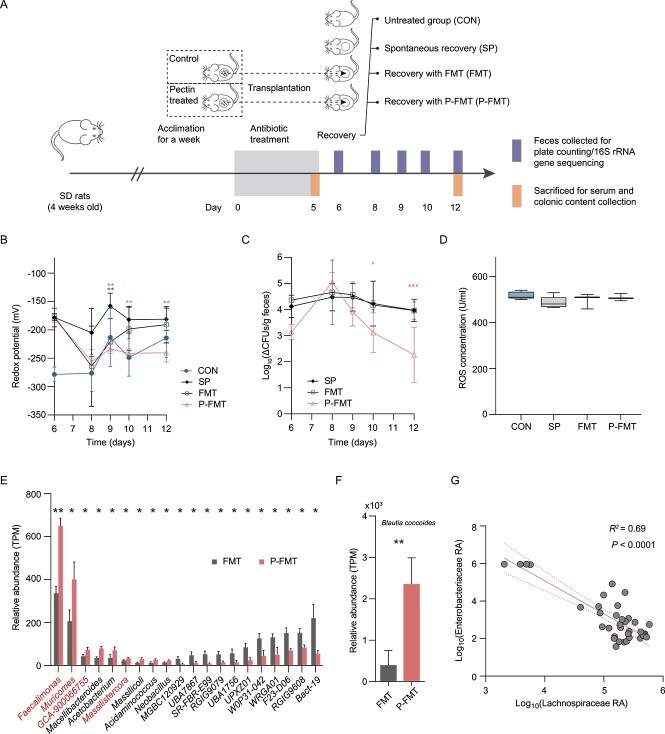
**Pectin-modulated gut microbiota improves post-antibiotic recovery from AID. A** Experimental design (*n* = 6). FMT/P-FMT rats are post-antibiotic rats receiving the fecal microbiota from control/pectin-treated donor rats, respectively. **B** Redox potential value in post-antibiotic rat feces. Asterisks indicate significance in fecal microbiota transplantation groups versus the SP group. One-way ANOVA, ^*^P < .05, ^*^^*^P < .01, ^*^^*^^*^P < .001. **C** Effects of FMT and P-FMT on the reduction of *Enterobacteriaceae* load after antibiotics treatment. Δ means the CFU of treated rats minus average of control rats. Asterisks indicate significance in fecal microbiota transplantation groups versus the SP group. **D** ROS concentrations in the serum of treated and control rats. **E** Abundance of significantly different genera (top 20) between FMT and P-FMT based on colon metagenome sequencing data (*n* = 3). One-way ANOVA, ^*^P < .05, ^*^^*^P < .01. TPM, transcripts per million. **F** The abundance of *Blautia coccoides* in the FMT and P-FMT colon microbiome (*n* = 3). One-way ANOVA, ^*^^*^P < .01. **G** the correlation between the *Lachnospiraceae* and *Enterobacteriaceae* based on Spearman correlation analysis. RA, relative abundance.

To determine whether it was the pectin-modulated gut microbiota that contributed to the PEC-accelerated post-antibiotic decrease of redox potential, we performed a fecal microbiota transplantation experiment. We validated the effect of the pectin-modulated microbiota on post-antibiotic recovery of the gut microbiome and ABX-elevated redox potential by transplanting the fecal microbiota of rats from the control and pectin-supplemented groups into post-antibiotic rats (Fig. 7A). P-FMT successfully accelerated post-antibiotic recovery from antibiotic-induced increase in gut redox potential. Fecal redox potentials of P-FMT rats were significantly lower than those of SP rats by the later timepoints of post-antibiotic recovery, and this situation was observed in P-FMT but not in FMT rats (Fig. 7B), indicating that pectin-modulated microbiota contributed to greater gut chemical environment recovery than FMT. When we monitored the quantity of *Enterobacteriaceae*, rats given P-FMT were also found to have a greater decrement in *Enterobacteriaceae* load with respect to SP rats by day 12, whereas no difference was observed between FMT and SP rats (Fig. 7C). ROS levels revealed no significant differences between comparisons of groups (Fig. 7D).

Metagenome sequencing generated a total of 1 572 611 534 reads, with a mean ± SEM of 98 288 221 ± 2 853 334 reads per sample (Table S11). After quality control and removal of host genes, a total of 1 538 313 322 reads were retained, with a mean ± SEM of 96 144 583 ± 2 818 146 per sample. After de novo assembly, 3 230 629 contigs were generated (N50 length of 4819 ± 389 bp), with a mean ± SEM of 201 914 ± 14 921 per sample. At the genus level, the major (top 20) differentially abundant genera between FMT and P-FMT rats were identified, including increased *Faecalimonas*, *Muricomes*, *GCA-900066755*, *Acetobacterium*, and *Massilistercora*, and decreased *Bact-19*, *RGIG9608*, and *F23-D06* post P-FMT (Fig. 7E). Specifically, the elevated genera dominantly belonged to the *Lachnospiraceae* family. In addition, P-FMT boosted the species *Blautia coccoides* and *Lachnoclostridium* sp. From the *Lachnospiraceae* family (Fig. 7F). Combining the data of all colon microbiomes, the abundance of *Lachnospiraceae* was significantly inversely correlated with that of *Enterobacteriaceae* (Fig. 7G, *R*^2^ = 0.69, *P* < .0001).

**Figure 8:**
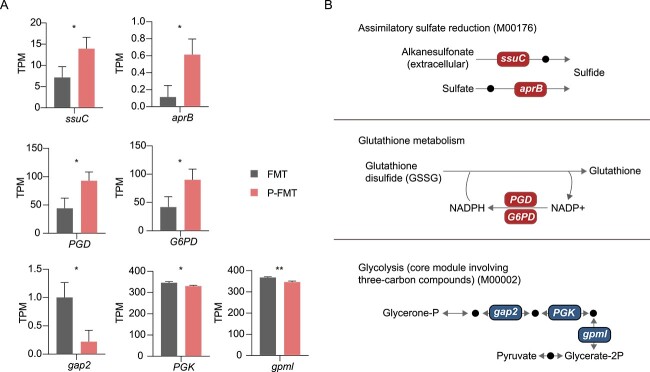
**Pectin-modulated gut microbiota changed oxidative and reductive metabolism in the colon microbiome of post-antibiotic recovery rats. A** Abundance of significantly different KO genes associated with oxidative- and reductive- metabolism between FMT and P-FMT colon metagenomes (*n* = 3). One-way ANOVA, ^*^P < .05, ^*^^*^P < .01. **B** KO genes appearing in **A** are shown in pathway modules modified from KEGG pathway maps like “assimilatory sulfate reduction”, “glutathione metabolism”, and “glycolysis”. Each box in a pathway represents a KO gene.

In KO analysis, significantly increased reductive metabolism–associated KO genes were identified in P-FMT that were involved in assimilatory sulfate reduction (M00176) and glutathione metabolism, including sulfonate transport system permease protein (*ssuC*/*K15554*), adenylylsulfate reductase subunit B (*aprB*/*K00395*), 6-phosphogluconate dehydrogenase (*PGD*/*K00033*), and glucose-6-phosphate 1-dehydrogenase (*G6PD*/*K00036*). In parallel, oxidative metabolism–associated KO genes involved in glycolysis (M00002) were decreased in P-FMT, including glyceraldehyde-3-phosphate dehydrogenase (*gap2*/*K00150*), phosphoglycerate kinase (*PGK*/*K00927*), and 2,3-bisphosphoglycerate-independent phosphoglycerate mutase (*gpml*/*K15633*) (Fig. 8A and B).

## Discussion

Antibiotic-induced gut dysbiosis is health-threatening [37, 38], and the recovery from this dysbiosis is often slow and incomplete [39]. This dysbiosis is associated with elevation in redox potential and blooms in the bacterial family *Enterobacteriaceae*. In our previous study, we observed that a pectin-enriched diet could decrease redox potential and modulate gut microbiome in pigs. Thus, in the current experiment, we constructed an antibiotics-induced gut dysbiosis rat model and explored the potential modulation effects of pectin supplementation on the gut chemical environment to improve post-antibiotic recovery from this dysbiosis. Here, it was found that pectin accelerated post-antibiotic reconstitution of gut microbiome composition and function orchestrated with reduced gut redox potential. Moreover, the pectin-modulated gut microbiota was identified to contribute to faster reduction of gut redox potential with enhancement on microbial reductive metabolism in post-antibiotic rats.

Antibiotic administration damages gut microbiota but allows aerobic microbes to thrive in a high–redox potential environment [40, 41]. To monitor dynamic changes in the gut micro-ecosystem after treatment with antibiotics, representative measurements (e.g., redox potential, pH, and *Enterobacteriaceae* load) in rat feces were evaluated in the experiment. This evaluation was aimed to timely confirm whether the AID model was well constructed, and get clues about whether pectin supplementation helps post-antibiotic recovery from AID. Our results demonstrated that this dysbiosis model was established successfully and demonstrated strikingly elevated redox potential and *Enterobacteriaceae* load after antibiotic treatment, effects that have also been observed previously in mice and humans [9, 10]. In addition, we found that PEC accelerated the reduction of Eh and *Enterobacteriaceae* in post-antibiotic recovery, which accorded with our hypothesis. Although pH was reported to be associated with Eh [42], fecal pH did not exhibit any significant responses to pectin treatment like those observed with Eh. This finding suggested that the pH of the gut environment was not the major contributor in enhancing post-antibiotic recovery by PEC.

To understand whether PEC accelerated gut microbial community reconstruction entirely from dysbiosis status in rats, we used 16S rRNA gene sequencing analysis to dissect the dynamics of microbiota reconstitution in rat feces in the post-antibiotic recovery process. The use of PEC was associated with faster restoration than SP from the sharply reduced level of the α-diversity of the gut microbiota. Many studies have consistently reported the ability of pectin to increase the bacterial α-diversity of the intestine [43]. The β-diversity results also indicated that PEC accelerated post-antibiotic microbial reconstitution in rat feces, and as the PCoA plot shows, the representative data points of PEC rats were closer to those of control rats than SP in each time point. To explore how a markedly disrupted gut microbiota was restored, microbial composition and function were taken into consideration [44]. The abundance and number of unrestored genera (or pathways) were summarized and exhibited lower levels in PEC than SP throughout the post-antibiotic process. To decipher the microbial contributor and associated functions during post-antibiotic recovery, the genera and pathways that were restored earlier in PEC were identified. Pectin supplementation favored the growth of strict anaerobic microbes from the *Butyricicoccaceae*, *Anaerovoracaceae*, and *Lachnospiraceae* families, which have the enzymatic repertoire to degrade pectin [45] and associate with attenuating diseases, such as inflammatory bowel disease (IBD) [46, 47]. Antibiotic treatment could increase the relative abundances of extensive microbial functions, such as glycolysis/gluconeogenesis, lysine degradation, and ascorbate and aldarate metabolism [48]. The restorations in predicted functional pathways advanced by PEC were related to glycolysis/gluconeogenesis and glutamine and glutamate metabolism. Glycolysis, a part of catabolic oxidative metabolism [49], was reported to be associated with the glucose diet, which was not protective for relieving high redox energy metabolism under post-antibiotic treatment [8]. The restoration and decrease in glycolysis by PEC suggested the improvement of pectin in the gut redox environment during post-antibiotic recovery.

Metagenome sequencing of colon contents was performed to assess the impact of pectin supplementation on post-antibiotic recovery in the large intestine, which is the key location for pectin fermentation and exerting benefits on gut health [50]. Similarly, pectin supplementation also accelerated the restoration of α-, β-diversity, composition and function in the colon microbiome. Anaerobic genera prefer a low redox potential environment [51], and their increase in abundance with pectin supplementation suggested that pectin was helpful for maintaining an anaerobic colon environment that coincides with the reduction of fecal redox potential in PEC rats. Gut microbial metabolism is associated with tolerance to antibiotics [52, 53] and ecological resilience after antibiotic treatment [54]. Considering changes in metabolic functions, antibiotic-activated catabolic oxidative metabolism was repressed and restored to the control level by PEC, suggesting an increase in resilience from dysbiosis. This observation agreed with previously reported findings that fiber repressed microbial catabolic oxidative metabolism and increased anabolic reductive metabolism to promote tolerance to antibiotics [8].

To further understand the shifts in oxidative and reductive metabolism, the changes of KOs were demonstrated and exhibited in a module representation by modifying the KEGG pathway reference maps. Accordingly, low abundance KOs involved in anabolic reductive modules, like sulfate-sulfur assimilation, glutathione biosynthesis, and the reductive pentose phosphate cycle, and high abundant KOs involved in catabolic oxidative modules, like glycolysis and histidine biosynthesis, were associated with SP, whereas they were restored to the control level by PEC. Assimilatory sulfate reduction by anaerobes generates reduced sulfur for biosynthesis processes [55], which is crucial for satisfying physiological requirements [56]. Glutathione plays an important role in regulating the luminal redox environment [22], and low levels of glutathione contribute to oxidative stress [57]. Collectively, these results indicated that pectin supplementation encouraged post-antibiotic recovery of gut microbial composition and function orchestrated with reduced redox potential and corresponding high redox microbial activities. In further research, antioxidants were screened out to intervene in gut redox potential, and we found that ferulic acid could modulate gut microbiota and decrease redox potential (Feng, et al, on 26 May 2024, unpublished). However, it is still difficult to unveil the causal relationship between gut microbiota and redox potential, and more researches are needed. Indeed, the gut ecosystem often exhibits disturbances accompanied by increased gut redox potential not only under antibiotics exposure but also in patients suffering from diseases like inflammation [58, 59], malnutrition [60], and obesity [61].. Hence, we suppose that pectin may also have the potential to alleviate these diseases through reducing redox potential.

Despite the contribution of pectin intervention in linking changes in gut redox potential on post-antibiotic gut microbiome reconstitution, the causal role of pectin on reduction of redox potential in post-antibiotic recovery is still unclear. Here, we investigated this underlying mechanism from different perspectives, including the antioxidant property of pectin, host response, and microbiota. To our knowledge, host-associated factors often impact gut redox potentials [22, 59]. For example, immune molecules like ROS or nitrate mediate redox activity during homeostasis and disease processes in the gastrointestinal tract [62, 63]. We focused on the colonic morphology, relevant electron acceptor (nitrate), and ROS levels, which might be representatives of overall host responses to pectin supplementation after antibiotics exposure. Our data demonstrated that PEC induced no significant changes in these features, suggesting that host-associated factors were not the major contributor to the PEC-induced reduction of redox potential.

A diet rich in antioxidants has been a strategy to improve redox balance to avoid oxidant conditions (e.g., inflammation) [64, 65]. The effects of this diet suggested the direct regulatory effects of antioxidant properties of dietary contents on gut redox status. Thus, the role of the antioxidant property of pectin itself on modulating gut redox potential could not be neglected. Our results indicated that all pectins with different antioxidant abilities could decrease redox potential in rat feces and had similar effects on microbiota composition in post-antibiotic recovery. The enrichments of obligate anaerobes both in the HP and LP groups hinted at the enhanced anaerobic environment of the gut lumen in post-antibiotic rats. These increased genera associated with gut health, including *Blautia*, *Akkermansia*, and *Turicibacter*, have been found to be associated with pectin fermentation [47, 66]. *Blautia*, a member of *Lachnospiraceae*, is a well-known butyrate producer and has shown attributable health benefits in intestinal diseases [67]. Besides, the genus *Akkermansia* is implicated as an enhancer of gut barrier function [66]. *Turicibacter* is reported as a bacterium with possible anti-inflammatory effects [68]. In short, these results proved that the acceleration in post-antibiotic decrease of redox potential by PEC could not be attributed to the antioxidant property of pectin.

Redox dynamics are linked to gut microbiota structure. To determine whether it was the pectin-modulated gut microbiota that encouraged the post-antibiotic decrease of redox potential, we carried out a fecal microbiota transplantation experiment. Pectin-modulated gut microbiota successfully accelerated the decrease of redox potential and *Enterobacteriaceae* load post-antibiotics, whereas the microbiota from control rats had no similar effects on them. Further analysis of the gut microbiota showed that anaerobic bacteria affiliated with the family *Lachnospiraceae* were significantly enriched in the P-FMT group compared with the FMT group, with similar results for pectin supplementation in the above experiments. Moreover, at the species level, we detected increased *B. coccoides* abundance in the P-FMT-treated rats. The depletion of *B. coccoides*, a strict anaerobe from the *Lachnospiraceae* family was reportedly associated with disease, such as intestinal inflammation [69], irritable bowel syndrome [70], and type I diabetes [71]. These findings suggested that an increase of *B. coccoides* might be beneficial for maintaining gut homeostasis and potentially improve restoration from dysbiosis. *Lachnospiraceae* abundance was negatively correlated with *Enterobacteriaceae* abundance in our study, suggesting the role of *Lachnospiraceae* in mitigating the proliferation of *Enterobacteriaceae* induced by antibiotic treatment [72]. In vivo, it was evidenced that a *Lachnospiraceae* isolate could partially restore colonization resistance against the antibiotic-induced aerobic pathogen *Clostridium difficile* [72], which supports our findings. The positively correlated relationship between the *Lachnospiraceae* members and maintaining of a low intestinal redox state has also been reported [73]. In the present study, anaerobic members of *Lachnospiraceae* family were enriched in PEC, HP, LP, and P-FMT groups, and may have made important contributions to improving post-antibiotic recovery from AID. This enrichment was consistently accompanied by improved signatures of low redox metabolic activities both in rats with pectin supplementation and pectin-modulated intestinal microbiota transplantation. Therefore, it was confirmed that pectin-modulated gut microbiota could accelerate post-antibiotic recovery from AID, a finding that was consistent with the observed effects of pectin supplementation. In addition, although we recognized the important role of *Lachnospiraceae* members on maintaining the homeostasis of gut microbiome redox functions, the underlying mechanism merits further investigation. For example, not all members within the *Lachnospiraceae* family exhibited increased abundances in response to pectin, and the inconsistent changes of *Lachnospiraceae* members in different experiments might relate to variability in gut microbiota composition among rats from different batches and relatively low repetitions, which were challenges for further research.

In the current study, redox potential was crucial for pectin to aid in gut microbial recovery post-antibiotic treatment, suggesting that the redox potential can act as a target for regulating gut microbiota or alleviating metabolic disorders with high gut redox potential (e.g., diarrhea and malnutrition). For example, a recent study demonstrated that reduction of gut redox potential could aid in host resistance to pathogen infection [74].

## Conclusions

In summary, our research demonstrated that pectin supplementation accelerated post-antibiotic gut microbiome reconstitution orchestrated with reduced gut redox potential. Moreover, the pectin-modulated gut microbiota was identified as a contributor to faster reduction of gut redox potential with enhancement of microbial reductive metabolism in post-antibiotic rats. This work reveals the potential of pectin to be a therapeutic aid for AID and highlights the important role of pectin-modulated intestinal microbiota on reducing redox potential in the post-antibiotic process. Our findings provide new insights into the mechanism of modulating gut redox potential by pectin and also identifying potential targets for improvement of the gut microbiota.

## Supplementary Material

Supplementary_material_1_wrae101

Supplementary_material_2_wrae101

Supplementary_information_Analytic_scripts_wrae101

## Data Availability

Sequencing data generated in the study were deposited into the NCBI Sequence Read Archive, with accession numbers PRJNA1039327, PRJNA1039509, PRJNA1039724, and PRJNA1041330. All scripts for statistics and figure generation in this study were available at https://github.com/xry11222/Xu-pectin_paper-code. The main data supporting the findings are available within this article and in the Supplementary Information files.
