# Supplementary material for: Pectin supplementation accelerates post-antibiotic gut microbiome reconstitution orchestrated with reduced gut redox potential
Source: ISME J. 2024 Jun 10;18(1):wrae101. doi: 10.1093/ismejo/wrae101 (PMC11203915; doi:10.1093/ismejo/wrae101)

**Fig. S1** **A** Average daily feed intake and **B** blood glucose levels in rats of CON, SP and PEC groups.

**
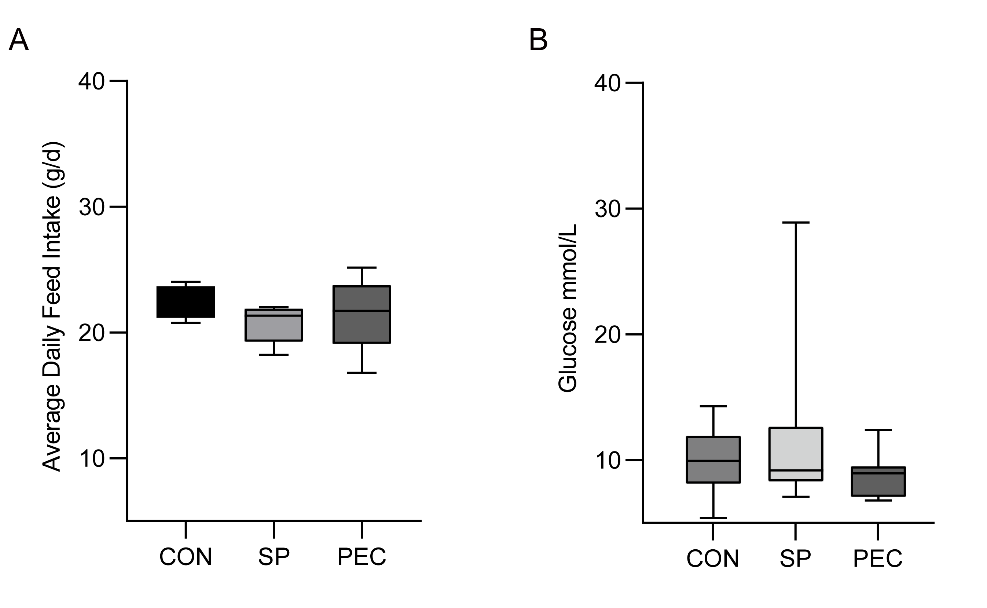
**

**Fig. S2** Relative abundances of six abundant phyla in rat colon microbiome. Asterisks indicate significant abundance differences between groups (*n* = 4 per group). One-way ANOVA, **P* < 0.05.


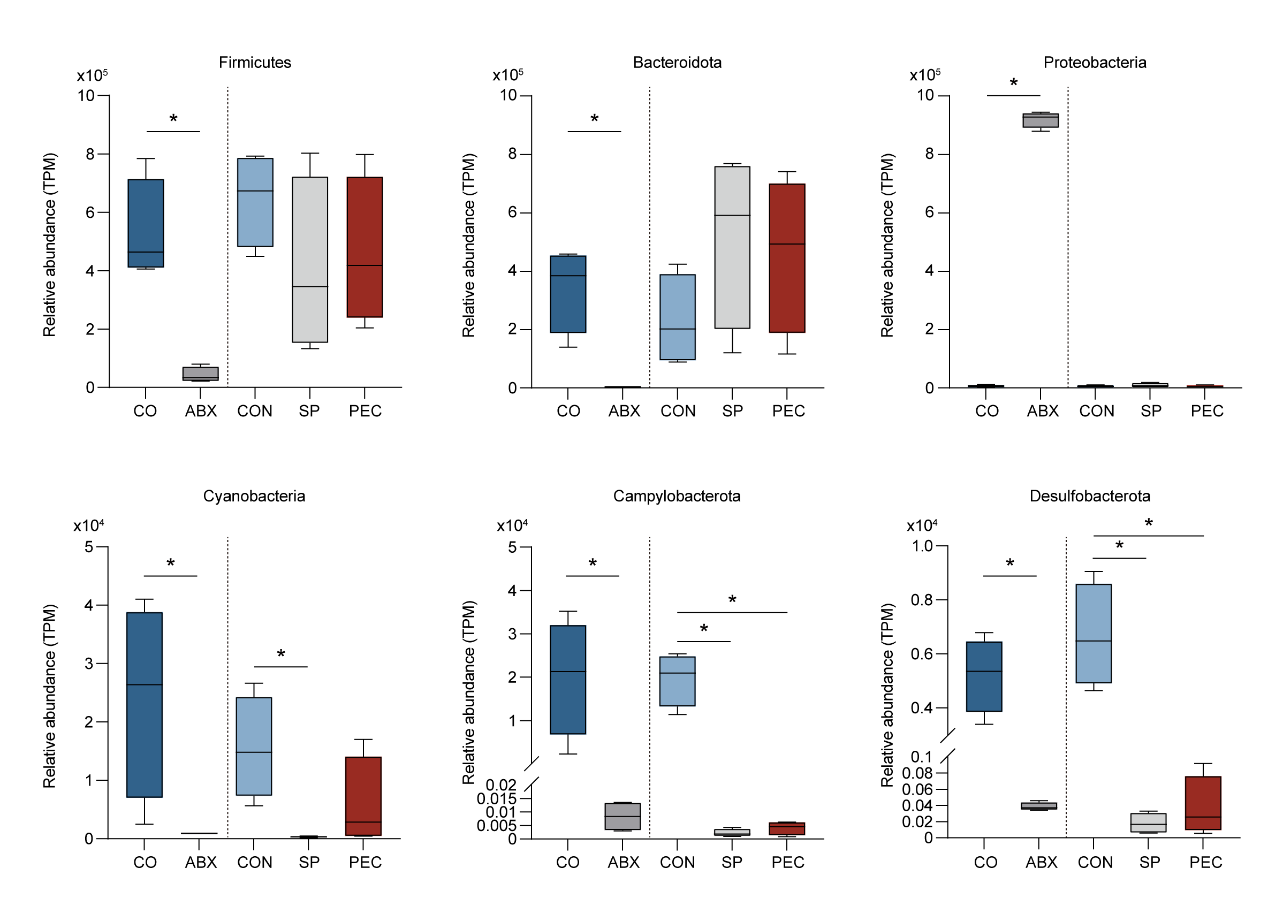


**Fig. S3** The network of the Spearman correlations between PEC-specific restored genera and species in colon and Enterobacteriaceae load in rat feces. Interactions with a *P* value < 0.05 are presented. The network was visualized by Gephi (v 0.9.7) software.


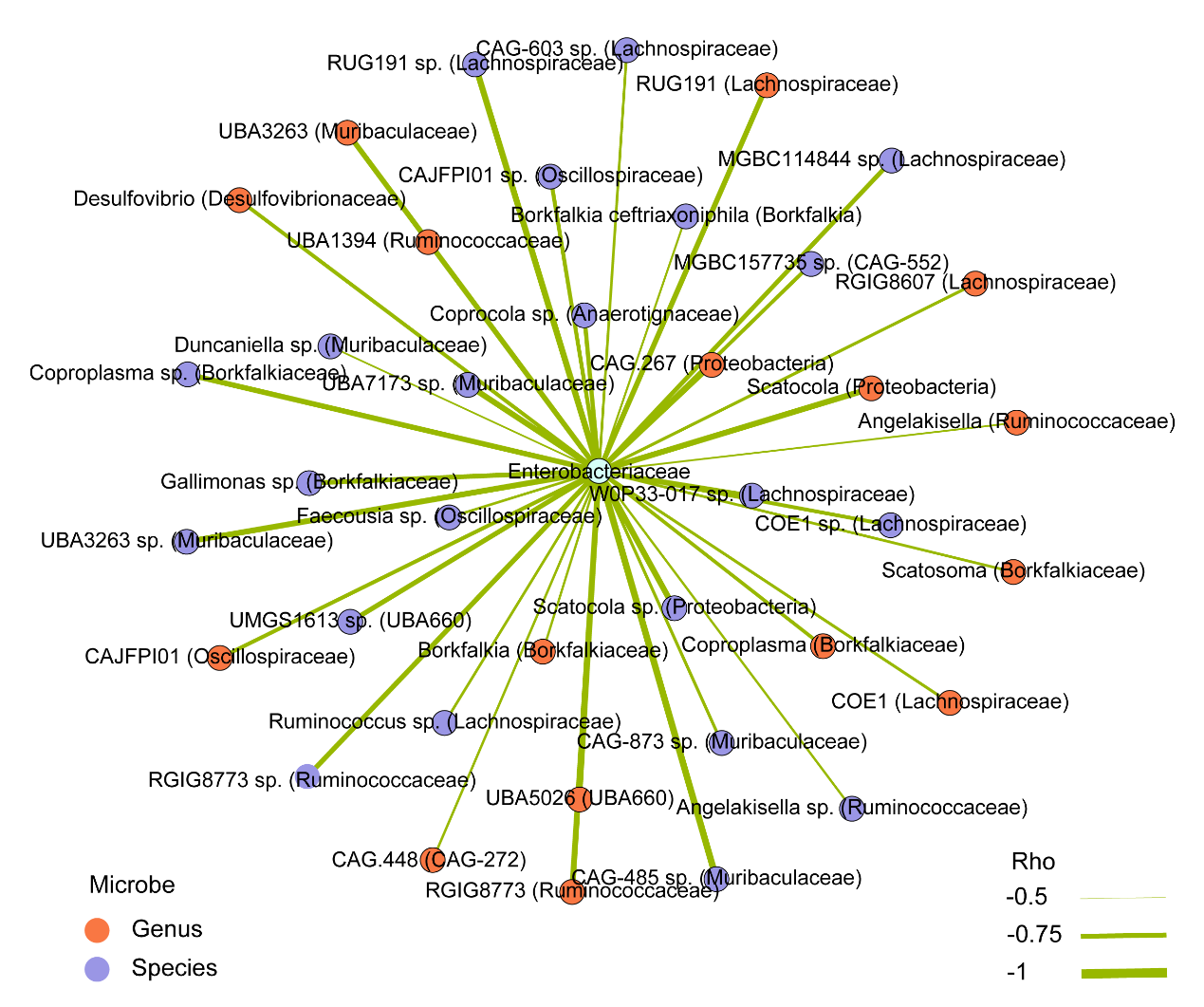


**Fig. S4** Pectin supplementation had no significant effects on electron transport chain (ETC) in colon microbiome during post-antibiotic recovery. Altered KOs by antibiotics administration involved in ETC including complex 1, succinate dehydrogenase / fumarate reductase, cytochromes and ATPase, are shown in the heatmap. The color indicates the relative abundance of the KO gene, which is normalized by z-score.


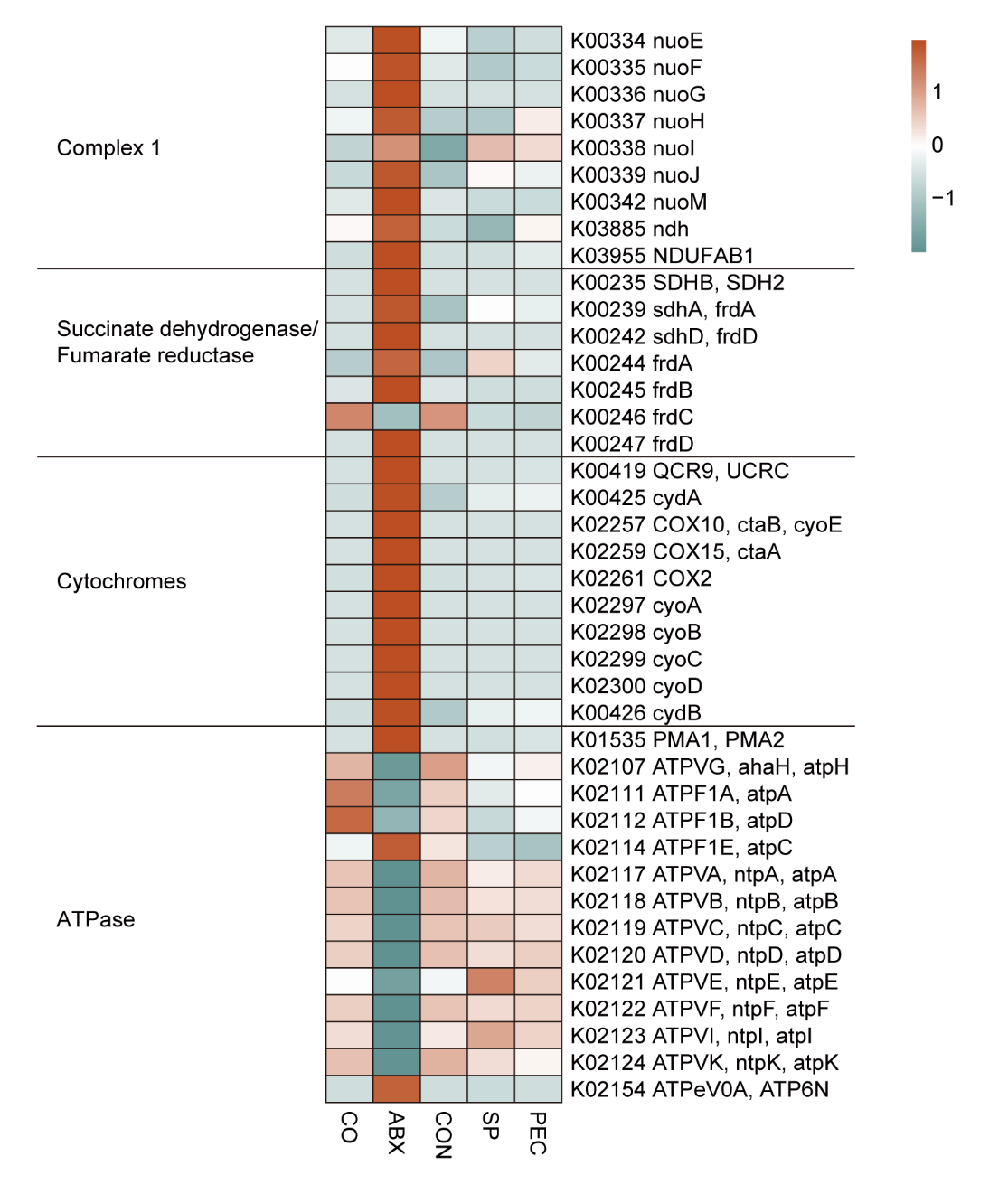


**Fig. S5** PEC had no significant effects on major host-associated factors involved in post-antibiotic recovery of redox potential in rats. (**A**) Representative images of hematoxylin and eosin-stained colonic sections and (**B**) cumulative histological scores. In magnified views, scale bar = 100 μm. (**C**) Electron acceptor (nitrate) level in the colon of treated and control rats. (**D**) ROS concentrations in the serum of treated and control rats. The statistics was performed with one-way ANOVA and post hoc by LSD.


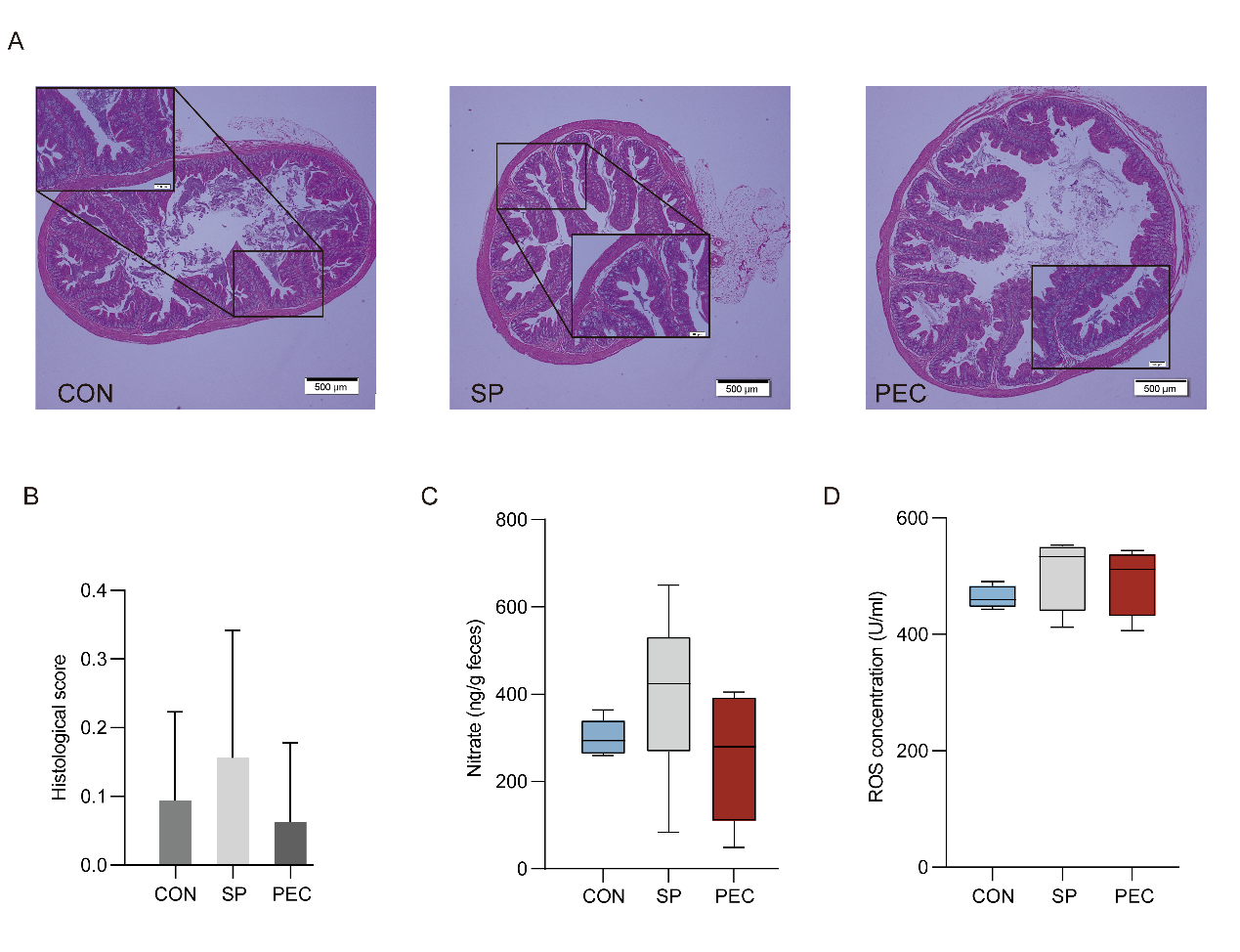

Supplement: Supplementary_material_2_wrae101 [file supplementary_material_2_wrae101.docx]
