# Supplementary material for: Pectin supplementation accelerates post-antibiotic gut microbiome reconstitution orchestrated with reduced gut redox potential
Source: ISME J. 2024 Jun 10;18(1):wrae101. doi: 10.1093/ismejo/wrae101 (PMC11203915; doi:10.1093/ismejo/wrae101)
Supplement: Supplementary_information_Analytic_scripts_wrae101 [file supplementary_information_analytic_scripts_wrae101.docx]

**Supplementary Information**

**Pectin supplementation** **accelerates post-antibiotic gut microbiome reconstitution** **orchestrated with reduced gut redox potential**

Rongying Xu^a, b^, Ni Feng^a, b^, Qiuke Li^a, b^, Hongyu Wang^a, b^, Lian Li^a, b^, Xiaobo Feng^c^, Yong Su^a, b*^, Weiyun Zhu^a, b^

^a^ Laboratory of Gastrointestinal Microbiology, Jiangsu Key Laboratory of Gastrointestinal Nutrition and Animal Health, College of Animal Science and Technology, Nanjing Agricultural University, Nanjing 210095, China

^b^ National Center for International Research on Animal Gut Nutrition, Nanjing Agricultural University, Nanjing 210095, China

^c^ Research Institute of General Surgery, Jinling Hospital, Nanjing University School of Medicine, Nanjing, China

**^*^Corresponding author**: Yong Su (E-mail: [yong.su@njau.edu.cn](mailto:yong.su@njau.edu.cn))

Main contents:

Scripts for statistics and figure generation

# Xu et al - paper – code

**16s pipeline includes:**

1. Filtering of reads；
2. 97% OTU clustering
3. Assigning taxonomy
4. Predict function using PICRUSt2

Details are as follows:

##16s rRNA pipeline:

**1. Filtering of reads；**

java -jar trimmomatic-0.36.jar PE -phred33 -threads 12 raw.R1.fq raw.R2.fq 341F-806R.trim.1.fq 341F-806R.s.1.fq 341F-806R.trim.2.fq 341F-806R.s.2.fq LEADING:0 TRAILING:20 SLIDINGWINDOW:10:20 MINLEN:75 2>341F-806R.trim.log

**2. 97% OTU clustering**

source activate qiime1

uparse -derep_prefix trim.fasta -output meta_derepprefix.fasta -sizeout 2> pipe.log

uparse -sortbysize meta_derepprefix.fasta -output meta_derepprefix_sorted.fasta -minsize 2 2>> pipe.log

pick_otus.py -i meta_derepprefix_sorted.fasta -o ./ -s 0.97 >>pipe.log

pick_rep_set.py -i meta_derepprefix_sorted_otus.txt -f meta_derepprefix_sorted.fasta -m most_abundant -o qiime_otu_rep.fasta >>pipe.log

uparse -usearch_global trim.fasta -db qiime_otu_rep.fasta -strand plus -id 0.97 -uc map.uc 2>> pipe.log

uc2otuseqids.pl -i map.uc -o otu_seqid.txt

pick_rep_set.py -i otu_seqid.txt -m most_abundant -f trim.fasta -o otu_rep.fasta

make_otu_table.py -i otu_seqid.txt -o otu_table.biom

biom convert -i otu_table.biom -o otu_table.txt --table-type "OTU table" --to-tsv

**3. Assigning taxonomy**

assign_taxonomy.py -m uclust -i otu_rep.fasta --similarity 0.8 -r silva.16s_bacteria.fasta -t silva.16s_bacteria.tax -o assign_taxonomy

**4. Predict function using PICRUSt2**

source activate picrust2

picrust2_pipeline.py -i otu_table.biom -s otu_rep.fasta -o PICRUSt2 --processes 20 --stratified --in_traits COG,EC,KO

##

**Metagenome pipeline includes:**

1. Filtering of reads；

- remove adaptor contaminations and low-quality reads
- remove host contaminations

1. Metagenomic DNA assemblies, gene predictions and construct non-redundant geneset;
2. Taxonomy assignment and KEGG annotation

Packages required:

a. Trimmomatic (v0.36)

b. BWA (v0.7.17)

c. MEGAHIT (v1.1.2)

d. Prodigal (v2.6.3)

e. CD-HIT (v4.6.7)

f. DIAMOND (v0.9.22)

**##Metagenome pipeline**: (use A-3-JS as an example)

**1. Filtering of reads；**

**##remove adaptor contaminations and low-quality reads**

java -jar -Xms20G -Xmx20G trimmomatic-0.36.jar PE -threads 8 -phred33 rawData/A-3-JS_R1.fq.gz rawData/A-3-JS_R2.fq.gz A-3-JS.clip.1.fq.gz A-3-JS.single.R1.fastq.gz A-3-JS.clip.2.fq.gz A-3-JS.single.R2.fastq.gz ILLUMINACLIP:/mnt/sdb/bin/Trimmomatic-0.33/adapters/merge.fa:2:30:10 SLIDINGWINDOW:4:15 MINLEN:75

**##remove host contaminations**

bwa mem -t 40 -M 00.ref/host.fa A-3-JS.clip.1.fq.gz A-3-JS.clip.2.fq.gz |awk '$3!="*"' > A-3-JS.host.sam

perl $Bin/remove-host.pl A-3-JS.host.sam A-3-JS

**2. Metagenomic DNA assemblies, gene predictions and construct non-redundant geneset;**

megahit -1 01.QC/A-3-JS.clean.1.fq.gz -2 01.QC/A-3-JS.clean.2.fq.gz --min-contig-len 500 -t 40 -o ./A-3-JS

prodigal -a A-3-JS.orf.faa -i /pathway/A-3-JS.contigs -f gff -o A-3-JS.gff -p meta -q -d A-3-JS.orf.ffn

cat $sampleid.orf.ffn >> all.fnn

cd-hit-est -i all.ffn -o uniqGeneSet.ffn -n 9 -c 0.95 -G 0 -M 0 -d 0 -aS 0.9 -r 1 -T 80

transeq -sequence uniqGeneSet.ffn -table 11 -trim -outseq uniqGeneSet.faa

bwa mem -t 40 -M -R \'\@RG\\tID:A-3-JS\\tSM:A-3-JS\\tLB:A-3-JS\\tPL:Illumina\\tPI:500\' uniqGeneSet.ffn A-3-JS.clean.1.fq.gz A-3-JS.clean.2.fq.gz > A-3-JS.sam

perl $Bin/uniqGene-ProfilebyBwa.pl uniqGeneSet.ffn A-3-JS.sam A-3-JS.sam.abd

**3. Taxonomy assignment and KEGG annotation**

#NR

diamond blastp -d /mnt/sdb/Database/NR/nr.all -q 06.profile/uniqGeneSet.faa -o uniqGeneSet.m8 -f 6 --evalue 0.00001 -k 10 -t ./ -b 8

#KEGG

source activate kegg

$Bin/kegg/kofamscan/bin/exec_annotation uniqGeneSet.faa -o kegg.txt -p $Bin/kegg/kofamscan/db/profiles/

Generation of figures:

The analytic scripts for alpha (Fig. 2A and Fig. 3A) and beta (Fig. 3B) analysis, PCoA (Fig. 2B) and PCA (Fig. 3C) analysis, Wilcoxon rank-sum test and correlation analysis (Fig. 7G and S3) were available at <https://github.com/xry11222/Xu-pectin_paper-code>.

Fig. 2C/E: After conducting differential analysis between groups by Wilcoxon rank-sum test in R software (v 4.2.1), genus or pathway in treated groups that significantly different from the control group in relative abundance was identified as “unrestored” genus or pathway. And the accumulative relative abundance of unrestored genera/pathways in treated groups were summarized within each timepoint during post-antibiotic recovery. The control groups for SP and PEC at D8 and D10 were the CON group at D6 and D12, respectively. The plots were visualized by GraphPad Prism (version 9).

Fig. 2D/F: Genera/pathways significantly altered by antibiotics in feces, which became indistinguishable to controls earlier in PEC than in SP, were shown in the heatmap. In the heatmap, relative abundances of genera/pathways were normalized by a z-score approach. The heatmap was plotted by https://www.bioinformatics.com.cn (last accessed on 10 May 2023), an online platform for data analysis and visualization.

Fig. 3G/H: Heatmap analysis of the restored genera/pathways specific to PEC. Each column in the heat map represents one group, and each row represents one genus/pathway. The color indicates the relative abundance of the genus/pathway, which is normalized by z-score. The heatmap was plotted by https://www.bioinformatics.com.cn (last accessed on 20 May 2023), an online platform for data analysis and visualization.
